# Influencing factors of health utility values in older adult people with normal cognition or mild cognitive impairment: a cross-sectional survey

**DOI:** 10.3389/fpubh.2025.1538665

**Published:** 2025-03-12

**Authors:** Yanqiu Du, Bingbing Zheng, Xiuru Wang, Tianlei Song, Di Liang, Jindong Ding Petersen, Jiayan Huang

**Affiliations:** ^1^Key Laboratory of Health Technology Assessment of National Health Commission, School of Public Health, Fudan University, Shanghai, China; ^2^School of Public Health, Hainan Medical University, Haikou, China; ^3^Section of General Practice, Research Unit for General Practice, Department of Public Health, University of Copenhagen, Copenhagen, Denmark; ^4^Research Unit for General Practice, Department of Public Health, University of Southern Denmark, Odense, Denmark

**Keywords:** mild cognitive impairment, health utility, mini-mental state examination, SF-6Dv2, depression

## Abstract

**Purpose:**

The purpose of this study is to present the findings of a cross-sectional survey on health state utility (HSU) values, a crucial metric for economic evaluations, and to analyze the primary factors influencing the HSU values of individuals with normal cognition (NC) or mild cognitive impairment (MCI).

**Methods:**

A community-based survey was conducted in Haikou City, China, employing cluster random sampling to select participants. The presence of NC and MCI was determined through the administration of the Chinese version of the Mini-Mental State Examination (MMSE). The assessment of HSU was conducted using the Chinese version of the Short Form Six Dimensions version 2 (SF-6Dv2), in conjunction with a questionnaire that collected data on socio-demographic characteristics, health-related behaviors, and health conditions. The HSU values were calculated using the SF-6Dv2 value set, which was developed for the Chinese population. A multiple linear regression model was constructed to identify the factors influencing HSU values.

**Results:**

The survey indicated that 536 older individuals were identified with NC (mean age 70.7, SD 7.1, 51.4% females), 245 were identified with MCI (mean age 73.0, SD 7.8, 67.4% females). The mean HSU values in NC group and MCI group were 0.792 (SD: 0.174) and 0.720 (SD: 0.199), respectively. The optimal multiple regression model for the MCI group demonstrated a linear relationship between age, depression symptomatology, and MMSE score with HSU, with coefficients of −0.009 (*p* < 0.001) for age and −0.132 (*p* < 0.001) for depression symptomatology. And for NC group, the optimal multiple linear regression model included five variables: age, sex, monthly personal income, depression symptomatology, and number of comorbidities.

**Conclusion:**

This study presented findings on HSU and its influencing factors in both the NC and MCI groups. The older adult individuals with MCI demonstrated lower HSU compared to their cognitively normal counterparts. The results of the factor analysis indicated that intervention programs designed to enhance the health-related quality of life for older adult individuals with MCI should include strategies to address depression.

## Introduction

Mild cognitive impairment (MCI) signifies an intermediary cognitive state that falls between the cognitive changes observed in the aging process and the criteria for diagnosing dementia (1). Around 15% of individuals with MCI progress to dementia within 2 years, with about 32% developing Alzheimer’s-related dementia within 5 years (2, 3). The study, based on Chinese data, estimated the prevalence of MCI among the older adult population in China is about 16%, with 38.8 million individuals affected by MCI in 2018 (4). As the Chinese population continues to age, it is estimated that there will be 29.5 million individuals aged 60 and above living with dementia by 2050. This is projected to have a significant impact on China’s population health and economy (5). It is therefore that interventions targeting individuals with MCI will become a critical public health strategy for early intervention.

Presently, early interventions for individuals with MCI primarily emphasize on non-pharmacological approaches, including cognitive training, physical exercise, dietary adjustment, and psychosocial interventions such as those targeting depressive symptoms (6, 7). Evidence from China indicates that cognitive training for individuals with normal cognition and those with MCI can help prevent or delay the decline in cognitive function (8). Compared to physical exercise and dietary intervention, cognitive training is still in a relatively developmental phase. Such programs typically require specific venues, specialized equipment, and professional support, resulting in a relatively higher average cost per participant. In urban areas with more advanced social welfare systems, such as Shanghai, cognitive training has been incorporated into public health service initiatives and is funded by the government (9). Nevertheless, despite its inclusion as a government procurement project, there is an absence of evidence regarding the economic value of cognitive interventions. This limitation precludes the possibility of conducting comparative analyses between cognitive training programs and other public health interventions, thereby impeding the ability of policymakers in other cities to make informed decisions regarding the incorporation of cognitive training into government-funded projects. Health state utility (HSU) is a crucial metric for economic evaluations. It provides a quantifiable measure of the value individuals place on different health states, enabling more accurate economic evaluations, cost-effectiveness analyses, and resource-allocation decisions in health-related research. However, a review of the existing literature reveals that HSU values for individuals with MCI in mainland China have not been reported (10).

This study aimed to measure HSU values using the Short Form Six Dimensions version 2 (SF-6Dv2) and to investigate associated factors in the older adult MCI population in Chinese communities. The reporting of HSU values will facilitate further research in other Chinese cities, providing a foundation for economic evaluations. Furthermore, the factors that influence HSU among older adult individuals with NC or MCI will be analyzed to inform the development of intervention programs designed to enhance the quality of life for this group.

## Methods

### Setting and sample

This study was conducted in Haikou City, the capital city of Hainan Province which located in the southmost part of China. Haikou City encompasses approximately 30% of the province’s older adult population (aged 60 and above) (11). This study employed a cluster random sampling method to select six communities out of 208 in Haikou City: Binlian, Jiahua, Binjiang, Yanfeng, Renmin, and Balun.

A community-based health status survey was conducted from April to July 2023. The survey team, in collaboration with the community health services centers (CHSC), recruited older adult individuals (aged 60 years and above) for the survey. When doctors at CHSC were conducting free physical examinations (funded by the government) for all older adult living in the target community, the survey team simultaneously carried out the recruitment work for this study. Exclusion criteria included those: (1) declining to provide informed consent; (2) with severe mental illness or other health issues preventing completion of the interview; and (3) not residing continuously in the designated communities for more than 6 months. Eligible participants provided informed consent and were interviewed face-to-face by trained investigators.

### Instrument

All investigators received a standard training and completed a simulation test conducted by the project management team encompassing neurologists and epidemiologists. The graduate students with professional backgrounds in clinical medicine or preventive medicine from Hainan Medical University (HNMU) served as the investigators. The training included research design, cognitive assessment tools, questionnaires, interview techniques, and data recording. Two neurologists from the Neurology Department of the Second Affiliated Hospital of HNMU provided the training on the Mini-Mental State Examination (MMSE). To ensure questionnaire quality, researchers performed immediate quality checks after collection, requesting clarifications as needed.

### MCI identification

MCI identification was based on the Chinese version of the MMSE, which has been widely used in medical or research institutions in China with solid reliability and validity performance among the Chinese population (12–14). The MMSE scores range from 0 to 30with lower scores indicating more severe cognitive impairment. The people with an MMSE score of less than 24 points and greater than 17 points are considered to have mild cognitive impairment (15).

### Health-related quality of life (HRQoL) measurement

The HRQoL was measured using the Chinese version of SF-6Dv2 (16). The SF-6Dv2, an improvement on SF-6D, comprises six dimensions: physical functioning, role limitations, social functioning, pain, mental health, and vitality (17). With five or six choice levels in each dimension, there are a total of 18,750 possible health states. For example, a person’s health status of 132354 indicated that the person selected the first level in the physical functioning dimension, the third level in the role limitations dimension, and the remaining numbers correspond to the selection levels in the remaining dimensions. The SF-6Dv2 is commonly used to calculate health utility values for non-communicable diseases in China (18–21), and also been used in the field of MCI research in other countries (ref). In this study, the HSU value set for the SF-6Dv2 developed among Chinese adults in eight provinces was used (22). The HSU value was obtained by subtracting coefficients in the value set of SF-6Dv2 for each dimension level of the health state from 1. For example, for the health state 132354, the utility value would be 1 – (PF1 + RL3 + SF2 + PN3 + MH5 + VT4) = 1 − (0 + 0.059 + 0.047 + 0.083 + 0.134 + 0.108) = 0.569. The coefficients corresponding to the different choice levels for each dimension can be found in Supplementary material. For example, the coefficient corresponding to “RL3” is 0.059. The value range of all health states for the general population is between −0.277 (the worst health state: 555655) and 1.000 (the best health state: 111111) (22).

### Associated factors

A questionnaire was developed to collect data on self-reported socio-demographic information (age, sex, education, and marital status), health-related behaviors such as drinking and smoking, living situation, Comorbid diseases such as hypertension, diabetes and stroke (Supplementary material), symptoms such as depressive symptomatology and hearing impairments, and living situations (4, 23, 24).

Depressive symptomatology was assessed using the 2-item Patient Health Questionnaire (PHQ-2) (23). This instrument is widely regarded as a suitable option for screening programs targeting older adults (24). Smoking was assessed as participants reported smoking status (yes/no), and if yes, information as being frequently exposed to passive smoking in the past week was obtained. Drinking was assessed as had any alcohol consumption in the past week (yes/no). Living situation was measured whether participants were living alone or not, determined as whether participants lived at home alone more than 5 days in the past week.

### Data collection

Before the survey, the trained interviewers informed the participants about the survey’s purpose and obtained informed consent. The MMSE and SF-6Dv2 survey was conducted by one-on-one questioning. The quality-of-life survey was completed by the participants, with assistance provided for those who were illiterate or visually impaired. The investigator checked questionnaire completion asked participants to supplement any missing answers on-site.

### Statistical analysis

Descriptive analysis for participants’ characteristics was performed. Counts and frequencies for MMSE scores were described. Differences in the HSU values across subgroups were assessed using one-way analysis of variance (ANOVA) when the grouping variables were normally distributed or Mann–Whitney *U* rank-sum otherwise (25, 26). Omega squared (*ω^2^*) was used as an effect size in ANOVA analysis to correct for the bias in the estimation of the amount of variance explained (27). Multiple linear regression was employed to examine factors influencing utility values, using a stepwise approach to optimize the model for covariates adjustment (28, 29). In the multiple linear analysis, age and MMSE scores were treated as continuous variables within the model. Binary variables (e.g., sex, marital status) were coded as 0 and 1, while ordinal variables (e.g., years of education, number of comorbidities) were represented by integer values. The details of variable recoding were provided in Supplementary material. Statistically significance was set at less than 0.05 (*p* < 0.05).

All statistical analyses were performed using the statistical software SPSS version 27.0 (IBM, Chicago, IL, United States).

### Ethics approval

This study was conducted in accordance with the World Medical Association’s Declaration of Helsinki. This study received ethical approval from the Ethics Committee of HNMU (HYLL-2022-301). Informed consent was obtained from all participants following detailed explanations of the study.

## Results

### Characteristics of participants

A total of 906 eligible older adults participated in the community survey. Screening with the MMSE identified 536 individuals with NC (normal cognition), 245 with MCI. The Cronbach’s alpha of the MMSE used in all older adult samples was 0.889. The Cronbach’s alphas of the MMSE in NC group and MCI were 0.785 and 0.921, respectively. In terms of MMSE scores, the people with a score of 25 was the largest in the NC group, accounting for 17.7% of the NC group (Table 1). In the MCI group, the people with a score of 18 was the largest, accounting for 21.6%, the people with a score of 21 was the smallest (11.4%).

**Table 1:** The distribution of the individuals by MMSE score.

NC		MCI	
MMSE score	*n* (%)	MMSE score	*n* (%)
30	73 (13.6)	23	30 (12.2)
29	67 (12.5)	22	37 (15.1)
28	85 (15.8)	21	28 (11.4)
27	80 (14.9)	20	45 (18.3)
26	80 (14.9)	19	52 (21.2)
25	95 (17.7)	18	53 (21.6)
24	56 (10.4)	–	–
*N*	536 (100.0)	*N*	245 (100.0)

NC, normal cognition; MCI, mild cognitive impairment; MMSE, Mini-Mental State Examination.

Table 2 presents the distribution of NC and MCI groups’ characteristics. The average ages of the NC and MCI group were 70.7 years (SD = 7.1) and 73.0 years (SD = 7.8), respectively. Among NC and MCI group, males accounted for 48.6 and 32.6%, 26.8 and 60.0% with a primary school education or less, 84.5 and 68.5% married, 59.8 and 74.6% had a monthly income below 2000 RMB, 5.7 and 11.8% lived alone, respectively. The above characteristics all showed significant differences between NC and MCI (*p* < 0.05).

**Table 2:** The characteristics of individuals.

	NC	MCI	*p*
*N*	536	245	
Age, years	70.7 (7.1)	73.0 (7.8)	<0.001
Age range, *n* (%)			0.002
60–69 years	250 (46.6)	96 (39.1)	
70–79 years	221 (41.2)	96 (39.1)	
≥80 years	65 (12.1)	53 (21.6)	
Men, *n* (%)	261 (48.6)	80 (32.6)	<0.001
Years of education, *n* (%)			<0.001
≤6 years	144 (26.8)	147 (60)	
7–9 years	222 (41.4)	67 (27.3)	
≥10 years	170 (31.7)	31 (12.6)	
Personal income^a^, *n* (%)			<0.001
≤2,000	321 (59.8)	183 (74.6)	
>2,000	215 (40.1)	62 (25.3)	
Marital status, *n* (%)			<0.001
Married	453 (84.5)	168 (68.5)	
Others^b^	83 (15.4)	77 (31.4)	
Way of residence, *n* (%)			0.003
Living alone	31 (5.7)	29 (11.8)	
Living with others	505 (94.2)	216 (88.1)	
Hypertension, *n* (%)	241 (44.9)	94 (38.3)	0.084
Diabetes, *n* (%)	83 (15.4)	36 (14.6)	0.775
Depressive symptomatology, *n* (%)	46 (8.5)	35 (14.2)	0.015
Comorbid diseases, *n* (%)			0.123
0	183 (34.1)	91 (37.1)	
1	210 (39.1)	108 (44)	
2	104 (19.4)	33 (13.4)	
≥3	39 (7.2)	13 (5.3)	
Smoking, *n* (%)	85 (15.8)	25 (10.2)	0.035
Drinking, *n* (%)	91 (16.9)	24 (9.7)	0.009

NC, normal cognition; MCI, mild cognitive impairment.

a CNY (Chinese Yuan) per month.

b Divorced, widowed, unmarried.

Regarding diseases, among the NC and MCI groups, the prevalence rates of hypertension were 44.9 and 38.3%, the prevalence rates of diabetes were 15.4 and 14.6%, and the depression screening rates measured by PHQ-2 were 8.5 and 14.2%, respectively (Table 2). Only the difference in the depression screening rate between NC and MCI group was significant (*p* = 0.015). Moreover, at the time of the survey, 34.1 and 37.1% of the older adult in the NC and MCI groups, respectively, reported having no diseases. In terms of habits, the proportions of people who smoke or drink are both higher in the NC group than in the MCI group, which were 15.8% versus 10.2% (*p* = 0.035) and 16.9% versus 9.7% (*p* = 0.009), respectively.

### HSU values among subgroups

The mean HSU values for NC group and MCI group, respectively, were 0.792 (95%CL, 0.777–0.807) and 0.720 (95%CL, 0.695–0.744). Table 3 showed the differences in HSU values among subgroups in NC group and MCI group. Univariate analysis revealed that in the NC group, factors significantly associated with HSU were age (*p* < 0.001), sex (*p* < 0.001), years of education (*p* = 0.002), income (*p* = 0.002), marital status (*p* = 0.022), hypertension (*p* = 0.047), depression (*p* < 0.001), and number of comorbidities (*p* = 0.002). However, in the MCI group, only age (*p* < 0.001), marital status (*p* = 0.038), and depression (*p* < 0.001) were significantly associated with HSU. In both the NC and MCI groups, no significant differences in HSU were observed between smokers and non-smokers or between drinkers and non-drinkers.

**Table 3:** Differences in health state utility values across subgroups in NC and MCI.

	NC	*p* (ES)	MCI	*p* (ES)
The mean of group (95%CL)	0.792 (0.777–0.807)	–	0.720 (0.695–0.744)	–
Age range (95%CL)		<0.001		<0.001
60–69 years	0.824 (0.803–0.845)	(0.038)	0.782 (0.752–0.813)	(0.082)
70–79 years	0.777 (0.756–0.798)		0.708 (0.666–0.751)	
≥80 years	0.716 (0.665–0.766)		0.624 (0.564–0.684)	
Sex (95%CL)		<0.001		0.338
Male	0.827 (0.810–0.844)	(0.039)	0.701 (0.650–0.753)	(0.000)
Female	0.758 (0.734–0.781)		0.727 (0.700–0.755)	
Years of education (95%CL)		0.002		0.141
≤6 years	0.750 (0.716–0.783)	(0.020)	0.699 (0.664–0.734)	(0.008)
7–9 years	0.797 (0.775–0.819)		0.755 (0.717–0.793)	
≥10 years	0.819 (0.796–0.842)		0.736 (0.661–0.812)	
Personal income^a^ (95%CL)		0.002		0.733
≤2,000	0.772 (0.752–0.793)	(0.016)	0.722 (0.692–0.751)	(−0.004)
>2,000	0.820 (0.799–0.841)		0.712 (0.661–0.762)	
Marital status (95%CL)		0.022		0.038
Married	0.799 (0.783–0.815)	(0.008)	0.737 (0.710–0.764)	(0.014)
Others^b^	0.751 (0.711–0.791)		0.680(0.626–0.734)	
Way of residence (95%CL)		0.221		0.255
Living alone	0.754 (0.677–0.831)	(0.001)	0.679 (0.599–0.760)	(0.001)
Living with others	0.794 (0.779–0.809)		0.724 (0.698–0.751)	
Hypertension (95%CL)		0.047		0.586
Yes	0.775 (0.751–0.798)	(0.005)	0.710 (0.674–0.746)	(−0.003)
No	0.805 (0.786–0.824)		0.724 (0.690–0.759)	
Diabetes		0.690		0.518
Yes	0.784 (0.744–0.825)	(−0.002)	0.739 (0.686–0.791)	(−0.002)
No	0.793 (0.777–0.809)		0.716 (0.687–0.744)	
Depressive symptomatology (95%CL)		<0.001		<0.001
Yes	0.666 (0.599–0.733)	(0.047)	0.600 (0.522–0.678)	(0.056)
No	0.803 (0.789–0.818)		0.739 (0.713–0.764)	
Comorbid diseases (95%CL)		0.003		0.601
0	0.813 (0.789–0.836)	(0.020)	0.735 (0.690–0.781)	(−0.005)
1	0.800 (0.777–0.822)		0.703 (0.667–0.740)	
2	0.767 (0.730–0.804)		0.737 (0.676–0.798)	
≥3	0.710 (0.642–0.778)		0.688 (0.568–0.808)	
Smoking (95%CL)		0.318		0.143
Yes	0.809 (0.776–0.842)	(0.000)	0.774 (0.699–0.850)	(0.005)
No	0.788 (0.772–0.805)		0.713 (0.686–0.739)	
Drinking (95%CL)		0.078		0.462
Yes	0.821 (0.790–0.852)	(0.004)	0.747 (0.679–0.816)	(−0.002)
No	0.785 (0.769–0.802)		0.716 (0.689–0.743)	

NC, normal cognition; MCI, mild cognitive impairment; ES, effect sizes.

a CNY (Chinese Yuan) per month.

b Divorced, widowed, unmarried.

The variations in HSU across different MMSE score groups (Table 4) were not statistically significant in the NC group (*p* = 0.053) or the MCI group (*p* = 0.082).

**Table 4:** The health utility value corresponding to the MMSE score.

NC				MCI	
MMSE score	HSU	*p*	MMSE score	HSU	*p*
30	0.770 (0.726–0.813)	0.053	23	0.643 (0.545–0.742)	0.082
29	0.741 (0.694–0.789)		22	0.707 (0.651–0.762)	
28	0.781 (0.743–0.820)		21	0.675 (0.577–0.773)	
27	0.802 (0.766–0.839)		20	0.760 (0.706–0.814)	
26	0.794 (0.754–0.834)		19	0.722 (0.673–0.771)	
25	0.818 (0.786–0.849)		18	0.757 (0.709–0.804)	
24	0.831 (0.791–0.871)				

NC, normal cognition; MCI, mild cognitive impairment; HSU, health state utility; MMSE, Mini-Mental State Examination.

### Multiple linear regression models for NC and MCI

For the NC group, the optimal multiple linear regression model included five variables: age, sex, monthly personal income (reference: lower-income group), depression symptomatology, and number of comorbidities (Table 5). Within this group, each additional year of age was associated with a 0.005 decrease in HSU (*p* < 0.001); HSU was 0.064 units lower in females compared to males (*p* < 0.001); HSU was 0.048 units higher in higher-income individuals than in those with lower incomes (*p* = 0.001); depression was associated with a 0.109-unit reduction in HSU compared to those without depression (*p* < 0.001); and each additional comorbidity was linked to a 0.023 decrease in HSU (*p* = 0.003).

**Table 5:** The multiple linear regression model of people with normal cognitive.

Variables	Beta coefficients	Standard error	*p* value
Constant	1.249	0.075	<0.001
Age	−0.005	0.001	<0.001
Sex (ref: Male)	−0.064	0.014	<0.001
Personal income (ref: lower group^a^)	0.048	0.014	0.001
Depressive symptomatology (ref: No)	−0.109	0.025	<0.001
Comorbid diseases (0–3)	−0.023	0.037	0.003

a Lower group, ≤2,000 CNY (Chinese Yuan) per month.

For the MCI group, the optimal model comprised three variables: age, depression, and MMSE score (Table 6). Among individuals in this group, each additional year of age was associated with a 0.009 decrease in HSU (*p* < 0.001); depression was linked to a 0.132-unit reduction in HSU; and within the MMSE score range of 18 to 23 (inclusive), each additional point was associated with a 0.015 increase in HSU (*p* = 0.023).

**Table 6:** The multiple linear regression model of people with mild impairment cognitive.

Variables	Beta coefficients	Standard error	*p* value
Constant	1.378	0.112	<0.001
Age	−0.009	0.001	<0.001
Depressive symptomatology (ref: No)	−0.132	0.033	<0.001
MMSE (18–24)	0.015	0.007	0.023

MMSE, Mini-Mental State Examination.

## Discussion

This study investigated the HSU among older adults with NC and with MCI in Chinese communities. Significant differences in HSU values based were observed based on age, marital status, living status, and depression. Although the mean HUS values for females and low-income MCI groups were lower than those for males and high-income groups, these differences were not statistically significant.

This study explored HSU and its influencing factors among older adult individuals with NC and those with MCI within Chinese communities. The HSU was found to be higher in the NC group compared to the MCI group (0.792 vs. 0.720, *p* < 0.001). Univariate analysis revealed significant differences in HSU related to age, sex, marital status, and depression symptomatology in both groups. While certain factors, such as sex, years of education, and income, showed significant differences in the NC group, these differences were not significant in the MCI group. Multiple linear regression analysis demonstrated a linear relationship between HSU and age, sex, income, depression symptomatology, and the number of comorbidities in the NC group. In the MCI group, the key factors were age, depression symptomatology, and MMSE score. The findings confirmed the disparity in HSU between the NC and MCI groups, highlighting both common and distinct influencing factors.

Currently, HSU values for MCI populations in developed countries have been reported in Canada (0.89), Germany (0.72), France (0.81), Netherlands (0.73), and United States (0.78) (30, 31). But there are no comparable data available for HSU values specifically for older adult Chinese individuals with MCI. Previous studies from China have indicated a significant link between cognitive impairment and decreased HRQoL, but they have not reported HUS values for the MCI group separately (32, 33). This may be due to the difficulty in collecting adequate samples of individuals with MCI compared to populations with moderate or severe cognitive impairments, the same challenge also noted in studies from Australia and Japan (34, 35). Although the reported HSU values were within a reasonable range compared to data from other countries, significant variability existed across different regions in China, and this study was limited to a provincial capital in southwestern China. Further research across diverse regions and various MCI populations were needed to better understand the causes of these differences.

Both univariate and multivariate analyses indicated that depression symptomatology is a significant influencing factor for HSU in both the NC and MCI groups. A study based on the French population had also demonstrated that depression is associated with poorer HRQoL in individuals with MCI (31). Consequently, interventions aimed at improving the quality of life for older adult individuals with MCI should consider incorporating psychological support for those experiencing depression (36).

Notably, while there was no significant correlation between HSU and MMSE scores in the NC group, a correlation was observed in the MCI group. This suggested that once cognitive function declines past a certain threshold, the deterioration in cognitive abilities becomes linearly related to reductions in HSU. Thus, cognitive interventions for the MCI population may enhance their HSU.

The results suggest that factors affecting MCI do not completely overlap with those influencing the HSU values in older MCI individuals. While smoking and alcohol consumption have been identified as risk factors for MCI according to previous studies (4, 37), this study did not find significant difference in HSU values between individuals with MCI who smoked or drank alcohol and those who did not. These results implied that traditional disease risk factors may no longer impact the quality of life for individuals who are already affected. Therefore, researchers should consider including additional factors beyond disease risks factors when assessing the quality of life for MCI patients.

This study had several limitations. First, due to its cross-sectional design, the associations between HSU and various factors cannot be interpreted as causal relationships. Second, community-based studies may overlook older adult individuals residing in hospitals and nursing homes, potentially leading to a healthier participant demographic compared to the broader older adult population. As such, future research should encompass studies of older adult populations in hospitals and nursing homes. In addition, in subsequent follow-up studies, data collection from more dimensions and sources needs to be considered. For example, measuring instrumental activities of daily living and collecting data from the perspective of caregivers (38, 39).

In conclusion, this study presented insights into HSU and its influencing factors in the NC and MCI groups in Chinese communities. Various factors were associated with participants’ quality of life, age and with depression significantly correlated with lower quality of life among older adults with NC and MCI.

## Data Availability

The raw data supporting the conclusions of this article will be made available by the authors, without undue reservation.
