# Supplementary material for: Influencing factors of health utility values in older adult people with normal cognition or mild cognitive impairment: a cross-sectional survey
Source: Front Public Health. 2025 Mar 12;13:1538665. doi: 10.3389/fpubh.2025.1538665 (PMC11936783; doi:10.3389/fpubh.2025.1538665)
Supplement: Supplementary file 1 [file Supplementary_file_1.docx]

**Supplementary Table 1:** The values corresponding to the different choice levels for each dimension

| Dimension and choice levels | Coefficient |
| --- | --- |
| Physical functioning (PF) |  |
| PF1 | 0.000 |
| PF2 | -0.038 |
| PF3 | -0.080 |
| PF4 | -0.140 |
| PF5 | -0.395 |
| Role limitation (RL) |  |
| RL1 | 0.000 |
| RL2 | -0.050 |
| RL3 | -0.059 |
| RL4 | -0.096 |
| RL5 | -0.097 |
| Social functioning (SF) |  |
| SF1 | 0.000 |
| SF2 | -0.047 |
| SF3 | -0.060 |
| SF4 | -0.093 |
| SF5 | -0.108 |
| Pain (PN) |  |
| PN1 | 0.000 |
| PN2 | -0.047 |
| PN3 | -0.083 |
| PN4 | -0.154 |
| PN5 | -0.388 |
| PN6 | -0.427 |
| Mental health (MH) |  |
| MH1 | 0.000 |
| MH2 | -0.033 |
| MH3 | -0.050 |
| MH4 | -0.132 |
| MH5 | -0.134 |
| Vitality (VT) |  |
| VT1 | 0.000 |
| VT2 | -0.029 |
| VT3 | -0.060 |
| VT4 | -0.108 |
| VT5 | -0.116 |

Source: Wu J, Xie S, He X, Chen G, Bai G, Feng D, Hu M, Jiang J, Wang X, Wu H et al: Valuation of SF-6Dv2 Health States in China Using Time Trade-off and Discrete-Choice Experiment with a Duration Dimension. PHARMACOECONOMICS 2021, 39(5):521-535.

**Supplementary Table 2:** The independent variable recoding

| Independent Variables and Original Values | Recoded Variable Values |
| --- | --- |
| Sex |  |
| Male | 0 |
| Female | 1 |
| Years of education |  |
| ≤6 years | 0 |
| 7-9 years | 1 |
| ≥10 years | 2 |
| Personal income ^a^ |  |
| ≤2000 | 0 |
| ＞2000 | 1 |
| Marital status |  |
| Married | 1 |
| Others ^b^ | 0 |
| Way of residence |  |
| Living alone | 0 |
| Living with others | 1 |
| Hypertension |  |
| Yes | 1 |
| No | 0 |
| Diabetes |  |
| Yes | 1 |
| No | 0 |
| Depression, n (%) |  |
| Yes | 1 |
| No | 0 |
| Comorbid diseases, n (%) |  |
| 0 | 0 |
| 1 | 1 |
| 2 | 2 |
| ≥3 | 3 |
| Smoking, n (%) |  |
| Yes | 1 |
| No | 0 |
| Drinking, n (%) |  |
| Yes | 1 |
| No | 0 |
| MMSE score (in NC model) |  |
| 30 | 6 |
| 29 | 5 |
| 28 | 4 |
| 27 | 3 |
| 26 | 2 |
| 25 | 1 |
| 24 | 0 |
| MMSE score (in MCI model) |  |
| 23 | 5 |
| 22 | 4 |
| 21 | 3 |
| 20 | 2 |
| 19 | 1 |
| 18 | 0 |

*NC, normal cognition; MCI, mild cognitive impairment; MMSE, Mini-Mental State Examination.*

*^a^ CNY (Chinese Yuan) per month*

*^b^ divorced, widowed, unmarried*

**Supplementary Table 3:** the distribution of

| Diseases | NC | MCI |
| --- | --- | --- |
| Heart disease | 65 | 22 |
| Hypertension | 241 | 94 |
| Diabetes | 83 | 36 |
| Hyperlipidemia | 68 | 25 |
| Stroke | 5 | 0 |
| Cerebral hemorrhage | 3 | 0 |
| Cancer | 0 | 1 |
| Others | 56 | 23 |

*NC, normal cognition; MCI, mild cognitive impairment.*

*Only when the respondents clearly state that they suffer from the above-mentioned diseases and indicate the time when they were diagnosed by a medical institution, or specify the names of the relevant treatments they have received or are currently receiving, or the names of the medications they are taking.*
